# Impact of physical decontamination methods on zirconia implant surface and subsequent bacterial adhesion: An in‐vitro study

**DOI:** 10.1002/cre2.486

**Published:** 2021-10-02

**Authors:** Nathan Chiang Ping Tan, Catherine M. Miller, Elsa Antunes, Dileep Sharma

**Affiliations:** ^1^ College of Medicine and Dentistry James Cook University Smithfield Queensland Australia; ^2^ College of Public Health, Medical and Veterinary Sciences James Cook University Smithfield Queensland Australia; ^3^ Australian Institute of Tropical Health and Medicine James Cook University Smithfield Queensland Australia; ^4^ College of Science and Engineering James Cook University Townsville Queensland Australia

**Keywords:** decontamination, dental implant, peri‐implantitis, zirconia

## Abstract

**Objective:**

To evaluate the effect of routinely used physical decontamination methods on the surface characteristics of zirconia implants and subsequent ability of bacteria to adhere in vitro.

**Background:**

Physical decontamination methods commonly used in peri‐implantitis therapy and routine implant maintenance can potentially alter zirconia implant surfaces.

**Methods:**

Acid‐etched zirconia discs were instrumented with titanium curette (TC), plastic curette, air abrasive device, ultrasonic scaler (US) with stainless steel tip. Following instrumentation, surface topography, and surface elemental composition was analyzed using 3D‐laser scanning microscopy and energy‐dispersive X‐ray spectroscopy, respectively. Subsequently, plaque biofilm was cultured on zirconia discs for 48 h and bacterial adhesion assessed using a turbidity test and scanning electron microscopy.

**Results:**

A significant difference in surface roughness was observed between the US and control group (*p* < 0.05). The US and TC caused gray surface discolouration on zirconia discs due to deposition of metallic residue as confirmed by X‐ray spectroscopy. No significant difference in bacterial adhesion was noted among all treatment groups (*p* > 0.05).

**Conclusion:**

TC and US with stainless steel tips should be used with caution due to deposition of metallic residue on the surface. Air abrasive devices and plastic curettes caused minimal surface alterations and are, therefore, safer for zirconia implant decontamination.

## 1 INTRODUCTION

Dental implants have become a well‐established treatment option to replace missing teeth in partially and completely edentulous patients (Stanford, 2007). It is estimated that more than 12 million dental implants are placed each year worldwide, significantly improving the quality of life for many individuals affected by the physical, social, and psychological impacts associated with tooth loss (Dosumu et al., 2014; Klinge et al., 2018; Sargozaie et al., 2017). The detrimental effects of edentulism include, but are not limited to, difficulties in eating and speaking, concerns about appearance, lowered self‐confidence, and feelings of bereavement (Dosumu et al., 2014).

Since the discovery of osseointegration in the late 1950s by Peri‐Ingvar Branemark, titanium implants have remained the gold standard in dental implantology (Guglielmotti et al., 2019; Klinge et al., 2018). Titanium implants are known for their high success rate owing to their excellent biocompatibility and favorable mechanical properties (Ozkurt & Kazazoglu, 2011). The main disadvantage of titanium as an implant material is its gray metallic appearance, which can be an aesthetic concern especially in the presence of thin gingival biotype or gingival recession (Apratim et al., 2015; Ozkurt & Kazazoglu, 2011; Sivaraman et al., 2018). It has also been reported that implant failure can occasionally occur due to the release of titanium ions into surrounding tissues, triggering a hypersensitivity reaction in susceptible patients (Kim et al., 2019). To overcome these drawbacks, zirconia implants have emerged as a viable alternative to titanium implants. Zirconia is a chemically inert material with minimal local and systemic side effects and is already extensively used in clinical dentistry for the fabrication of crowns, bridges, and implant abutments (Grech & Antunes, 2019; Munro et al., 2020). Zirconia is also a highly biocompatible material with an aesthetically pleasing tooth‐colored appearance, acceding to the increasing demand for metal‐free dental implants (Grech & Antunes, 2020; Ozkurt & Kazazoglu, 2011).

Much like natural teeth, dental implants are susceptible to developing diseases and complications. According to the 2017 World Workshop Classification of Periodontal and Peri‐implant Disease and Conditions (Caton et al., 2018), two types of peri‐implant disease known as peri‐implant mucositis and peri‐implantitis exist. Peri‐implant mucositis is a reversible inflammatory condition affecting the soft tissues surrounding an implant and is characterized by redness, swelling and bleeding (Caton et al., 2018). If left untreated, peri‐implant mucositis can progress to peri‐implantitis which involves the irreversible and progressive destruction of peri‐implant bone (Caton et al., 2018). Peri‐implantitis is one of the main causes of implant failure and is estimated to affect up to 18.8% of implant patients (Atieh et al., 2012). Routine supportive periodontal care is crucial in the prevention and management of peri‐implant disease (Gulati et al., 2014; Khan et al., 2020; Renvert et al., 2019).

Various instruments have been proposed for implant maintenance and peri‐implantitis therapy, including the use of metal and plastic curettes, ultrasonic scalers, air abrasive devices, prophylaxis cups, and laser systems (Gulati et al., 2014; Khan et al., 2020; Khan & Sharma, 2020; Louropoulou et al., 2012). However, some of the currently used decontamination methods can roughen implant surfaces, creating niche environments for bacterial colonization which in turn, increases the risk of peri‐implant disease (Louropoulou et al., 2012; Yeo et al., 2012). As such, physical decontamination methods should not only be effective in removing plaque and calculus but also safe in terms of preventing surface alterations and biocompatibility issues (Louropoulou et al., 2015).

To date, studies have primarily focused on instruments for decontamination of titanium implants and little is known about their suitability for zirconia. Hence, the primary aim of this in‐vitro study was to determine the effects of various physical decontamination methods on the surface characteristics of zirconia implant surface. The secondary aim was to assess changes in bacterial adhesion on treated zirconia surfaces following instrumentation. Our null hypothesis was that the physical decontamination methods tested would not alter the surface characteristics of the yttria‐tetragonal zirconia discs and, therefore, there would be no change in bacterial adhesion after treatment.

## 2 MATERIALS AND METHODS

### 2.1 Sample preparation

Yttria‐tetragonal zirconia polycrystal (Y‐TZP) discs measuring 16 mm in diameter and 3 mm in thickness were fabricated by uniaxial pressing and sintering commercial 3 mol% yttria‐partially stabilized zirconia powder (70% tetragonal, 30% monoclinic) using the protocol described in Munro et al. (2020). Y‐TZP discs were then immersed in 40% hydrofluoric acid (Scharlab, Barcelona, Spain) for 30 min to create an acid‐etched zirconia implant surface before being rinsed with purified water to remove any remaining acid or residue on the surface.

### 2.2 Cleaning procedure

Twenty acid‐etched Y‐TZP discs were equally and randomly divided into five treatment groups based on the type of instrument being examined. These included control (untreated samples), Titanium curette (Langer 1/2, Hu‐Friedy Mfg. Co. LLC, USA), Air abrasive device (Prophy‐mate NEO, NSK, Australia) with glycine powder (Perio‐mate, NSK, Australia) plastic curette (Implacare II, Hu‐Friedy Mfg. Co. LLC, USA) and Piezoelectric ultrasonic scaler with stainless steel tip (Suprasson P5 Satelec, Acteon, France).

Individual discs were oriented horizontally on a flat table and manually stabilized to prevent movement during treatment. All cleaning procedures were performed by an experienced dental clinician (N.T.).

### 2.3 Titanium curette and plastic curette

Fifty overlapping strokes were performed along the entire surface of each sample using the cutting edge of the curette. Moderate finger pressure was applied with the aim of replicating the amount of force normally used in clinical practice to remove calculus from an implant surface. A new curette was used for each sample to ensure that instruments were sharp prior to use.

### 2.4 Air abrasive device

The air abrasive device (AA) was loaded with glycine powder to the recommended level according to the manufacturer's instructions before being applied onto each sample. The AA was moved steadily over the entire surface for 1 min with the nozzle directed perpendicular to the sample at a distance 0.5 cm to 1 cm away.

### 2.5 Ultrasonic Scaler

The water coolant supply on the ultrasonic scaler (US) (Suprasson P5 Satelec, Aceton, France) was adjusted to a level consistent with routine use in clinical practice and confirmed via visual inspection. The working lateral surface of the US tip was applied for 1 min at 70% power setting on each sample.

Following instrumentation, all samples were wiped with minimal pressure using a lint‐free cloth soaked with 70% ethanol to remove debris and contaminants before being dried.

### 2.6 Laser scanning microscopy

Surface characterization of three Y‐TZP discs from each treatment group was performed using laser scanning microscopy (LEXT OLS4100, Olympus Corporation, Japan). Three scanned areas, each measuring 1.29 × 1.28 mm in dimension, were randomly selected on each sample for surface measurements. These measurements were carried out using a Gaussian filter, a low‐pass smoothing filter designed to reduce noise and separate roughness from waviness and form (Munro et al., 2020). The following parameters were chosen to provide information related to various facets of surface topography:

- Sa (μm): mean surface roughness; measure of arithmetical mean height
- Sz (μm): maximum surface height; sum of the highest peak and lowest valley
- Sku (units): kurtosis; measure of sharpness of the surface height distribution
- Ssk (units): skewness; measure of symmetry about the mean reference plane

Representative two‐dimensional (2D) and three‐dimensional (3D) laser scanning microscopy images (10× magnification) of samples in each treatment group were then acquired.

### 2.7 Scanning electron microscopy/Energy‐dispersive X‐ray spectroscopy (SEM‐EDS)

To determine the elemental composition of zirconia discs following treatment, one Y‐TZP disc from each treatment group was coated with a thin layer of carbon. An SEM (JSM‐5410LV, Jeol, Japan) equipped with an EDS detector (Oxford instruments, X‐Max detector, Oxford, UK) was used for surface analysis. EDS analysis was performed in three randomly selected points on each sample to detect and quantify the elemental composition of the zirconia discs before and after each treatment.

### 2.8 Bacterial adhesion assay

Following surface analysis, Y‐TZP discs were wiped with 70% ethanol and autoclaved at 134°C for 3.5 min in a steam sterilizer. The discs were placed into individual wells of 12‐well cell culture plates in preparation for bacterial adhesion assay using a protocol adapted from Park et al. (2015).

After ethical approval was obtained from the James Cook University Human Research Ethics Committee (#H8260), pooled saliva was collected from healthy participants with no active dental disease or known medical conditions (*n* = 5) and centrifuged at 1500*g* for 10°min to remove debris. The supernatant containing salivary bacteria was collected and diluted in a 1:2 ratio with Todd‐Hewitt Broth growth medium. A 5 mL aliquot of undiluted supernatant was centrifuged further at 8000*g* for 10 min to retrieve salivary glycoproteins essential for bacterial adherence. The supernatant containing the glycoproteins was removed and a 250 μL aliquot carefully dispensed onto each disc. The glycoproteins were allowed to attach for 30 mins to form an acquired pellicle. Subsequently, 2 mL of saliva/growth medium was added to each well containing a Y‐TZP disc before being incubated at 37°C for 48 h. Following incubation, the saliva/growth medium was removed and discs rinsed with phosphate‐buffered saline (PBS; pH 7.4) to remove any unattached bacteria. 1 mL of PBS was added to each well and discs sonicated for 10 min to detach adhered bacteria into the solution. The solution from each well was then aliquoted in triplicate into a 96 well cell culture plate. The number of bacteria present in each sample was estimated by determining optical density (OD_600_) in a microplate absorbance reader (iMark Microplate Absorbance Reader, Bio‐Rad Laboratories Inc, CA, USA).

### 2.9 Scanning electron microscopy

Qualitative analysis of bacterial adhesion on treated Y‐TZP discs was conducted using scanning electron microscopy (SEM) (Phenom™ G2 pro, Phenom‐World BV, Netherlands). Bacteria were grown on Y‐TZP discs for 48 h using the protocol described above. After rinsing, attached bacteria were fixed by immersion in 3% glutaraldehyde for 15 min followed by dehydration in graded concentrations of ethanol (25%, 50%, 75%, 95% and 100% ethanol for 5 mins at each concentration). The discs were then immersed in a 1:1 solution containing ethanol and hexamethyldilazane (HMDS) for 15 min followed by 100% HMDS for 5 min before being left to dry inside a fume hood for 24 h. The samples were mounted onto aluminum stubs using conductive carbon tabs before being sputter‐coated with gold (Spi‐Module™ Sputter Coater, SPI Supplies, USA) prior to SEM evaluation. Three areas on each sample were randomly selected for bacterial adhesion evaluation at 10,000× magnification.

### 2.10 Statistical analysis

Statistical analysis of data was performed using GraphPad 8.4 (GraphPad Software, CA, USA). Data related to surface parameters (Sa, Sz, Sku, and Ssk) and optical density (OD) was expressed as mean ± standard error measurements and analyzed using one‐way ANOVA. The post‐hoc Tukey test was used for multiple comparisons between groups. A *p*‐value <0.05 was considered statistically significant.

## 3 RESULTS

### 3.1 Surface morphology

**Figure 1:**
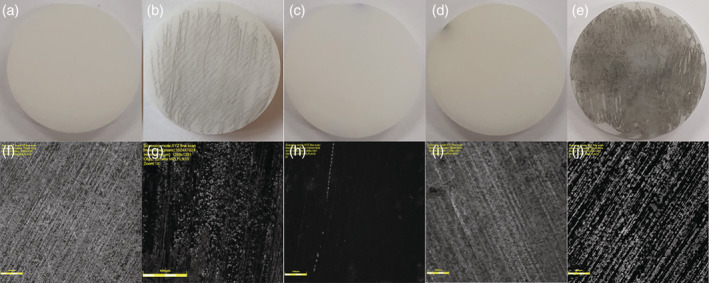
Changes to the surface of zirconia discs are visible after instrumentation. Discs were treated with instruments before being visually inspected and analyzed by laser scanning microscopy. (a–e) Representative photographic images of acid‐etched Y‐TZP samples following instrumentation (a) no treatment; (b) titanium curette (TC); (c) air abrasive device (AA); (d) plastic curette (PC); (e) ultrasonic Scaler (US). (f–j) Representative 2D laser scanning microscopy images at 10× magnification (f) control; (g) titanium curette (TC); (h) air abrasive device (AA); (i) plastic curette (PC); (j) ultrasonic scaler (US)

The surface morphology of Y‐TZP samples following instrumentation with the US, AA, TC, and plastic curette (PC) is shown in Figure 1. Visual inspection of Y‐TZP discs showed that surfaces treated with AA (Figure 1c) and PC (Figure 1d) had a similar morphology to untreated discs (Figure 1a) with no visible signs of surface alterations. In contrast, gray discolouration was seen on surfaces treated with TC (Figure 1b) and US (Figure 1e) in the form of numerous metallic marks. Due to these metallic marks, the US treated surface showed high irregularity (Figure 1g) and TC treated surface (Figure 1j) was darker in comparison to the control. In PC treatment and control groups (Figure 1f, i), multiple linear striations running obliquely across the surface were essentially created during the manufacturing process. These manufacturing lines were not visible on surfaces treated with AA (Figure 1h).

### 3.2 Surface topography

**Figure 2:**
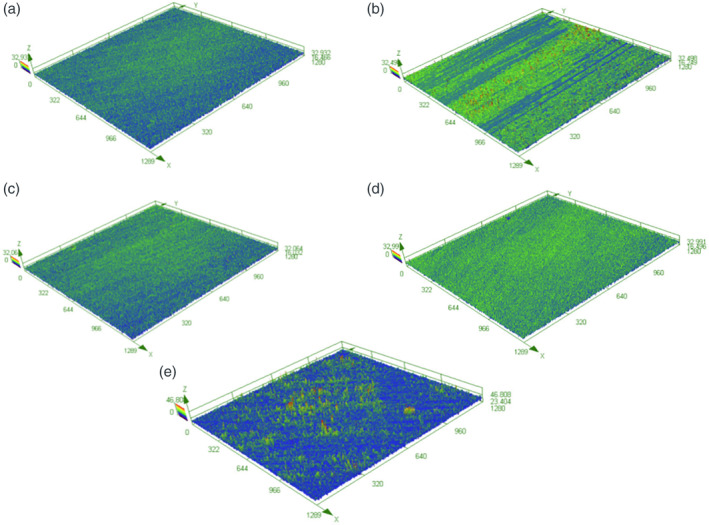
Three‐dimensional laser scanning microscopy reveals differences in surface morphology after treatment. Images were obtained at three randomly selected sites using digital laser scanning microscopy and representative wireframes were generated. Wireframes are shown in micrometers (μm.) A‐E representative images (10X magnification) of acid‐etched Y‐TZP samples following (a) no treatment (b); titanium curette (TC); (c) air abrasive device (AA); (d); plastic curette (PC); (e) ultrasonic Scaler (US)

**Figure 3:**
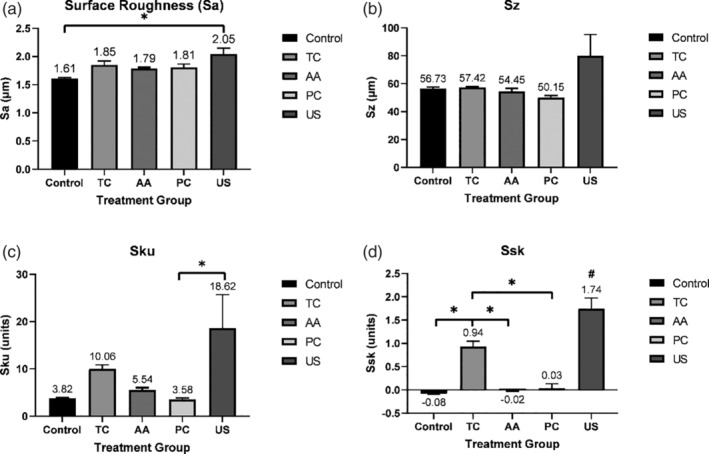
Results of the topographical analyses by laser scanning microscopy on acid‐etched zirconia discs after treatment. Surface parameters indicative of changes in surface morphology were determined for treated Y‐TZP samples using 3D laser scanning microscopy. (a) Sa, arithmetic mean height; (b) Sz, maximum surface height; (c) Sku, kurtosis; (d) Ssk, skewness. Data is presented as mean ± standard error (3 sites per disc). Titanium curette (TC); air abrasive device (AA); plastic curette (PC); ultrasonic Scaler (US). * indicates *p* < 0.05 between two treatment groups according to post hoc Tukey test; # indicates *p* < 0.05 with all treatment groups

Three‐dimensional surface characterization of untreated acid‐etched Y‐TZP samples revealed a relatively smooth and homogenous surface (Figure 2a). Similar homogeneity was observed on the discs treated with air abrasive (Figure 2c) and plastic curette (Figure 2d). Discs treated with TC (Figure 2b) or the US (Figure 2e) had more heterogenous surfaces with evidence of debris located on the surface. Topographical analysis of surface parameters showed acid‐etched discs to have a mean Sa measure of 1.6 μm (Figure 3a). Of the four treatments, only discs treated with the US had a Sa measurement that was significantly greater than untreated discs (Figure 3a). Similarly, discs treated with the US had a significantly higher measurement in Sku compared with discs treated with the plastic curette (Figure 3c). When Ssk was examined, discs treated with TC or US both showed a significant increase compared with untreated and discs treated with air abrasive or plastic curettes (Figure 3d). No significant difference in Sz was observed between untreated and treated discs (Figure 3b). The prevalence of peak‐like structures on surfaces treated with US and TC (Figure 2b, e) can be attributed to the presence of metallic remnants from the abraded instrument tips.

### 3.3 Energy dispersive X‐ray spectroscopy

The results of the Energy dispersive X‐ray spectroscopy (EDS) are shown in Table 1. Analysis revealed that all zirconia discs had a relatively high proportion of zirconium (Zr) and oxygen which are constituents of zirconium dioxide (ZrO_2_), small amounts of yttrium (Y) that is the dopant used to partially stabilize the zirconia, along with minor traces of hafnium (Hf). The control, AA, TC, and PC groups had fluorine (F) possibly due to the use of 40% hydrofluoric acid in the preliminary phase of the study. The elemental composition of US treated surfaces, unlike other treatment groups, included chromium (Cr) and iron (Fe) which are metallic elements commonly found in stainless steel. The deposition of metallic remnants, titanium (Ti), and barium (Ba), was also observed on TC treated surfaces. In terms of AA, unusual traces of gallium (Ga) and osmium (Os) were found along with calcium (Ca) and potassium (K) which are likely due to residual glycine powder remaining on the surface. Low levels of potassium (K) and sodium (Na) were also detected on surfaces treated with PC.

**Table 1:** Elemental composition (mean Wt%) of treated Y‐TZP surfaces analyzed (three sites per disc) using energy dispersive x‐ray spectroscopy (EDS). Titanium curette (TC); air abrasive device (AA); plastic curette (PC); ultrasonic Scaler (US)

	Control	TC	AA	PC	US
Zr	68.56	68.70	61.23	62.97	67.72
O	25.08	25.50	23.31	23.65	25.60
Hf	1.46	1.52	1.47	1.27	1.42
F	2.06	0.75	8.27	7.27	–
Y	2.84	2.58	5.30	4.09	2.23
Ti	–	0.59	–	–	–
Cr	–	–	–	–	0.63
Fe	–	–	–	–	2.40
Ga	–	–	0.10	–	–
Ba	–	0.36	‐	–	–
Os	–	–	0.04	–	–
Na	–	–	–	0.53	–
Ca	–	–	0.19	–	–
K	–	–	0.09	0.22	–

### 3.4 Bacterial adhesion assay

**Figure 4:**
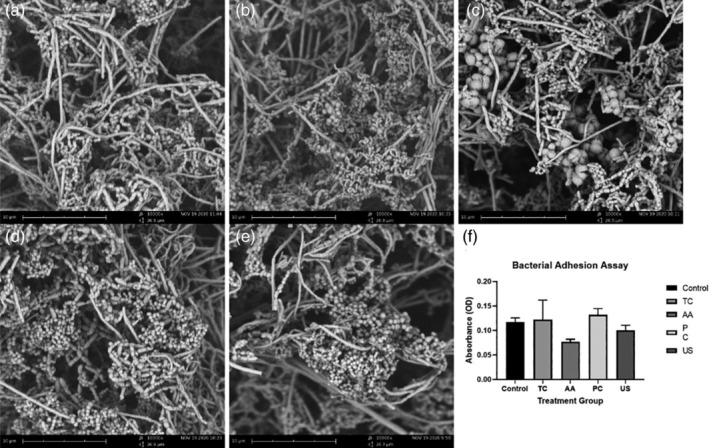
Formation of biofilms on acid‐etched zirconia surfaces is not affected by instrumentation. Biofilms of salivary bacteria were established on zirconia discs as described in the materials and methods. After 48 h incubation, non‐adhered bacteria were washed away and attached bacteria visualized by SEM (a–e). Spherical‐shaped bacterial cells intertwined within a dense network of extracellular matrix are visible. Representative images (10,000× magnification) are shown for each instrument used. (a) Untreated (b) titanium curette (TC); (c) air abrasive device (AA); (d) plastic curette (PC) (e) ultrasonic scaler (US). In separate experiments attached bacteria were dislodged and numbers estimated by measurement at OD600 (f). Results are presented as mean ± standard error, (three discs per group)

Bacterial adhesion was estimated by measurements of OD_600_ (optical density) of dislodged bacteria. No significant difference in OD_600_ was observed between any of the treatment groups compared with the untreated control (Figure 4f; *p* > 0.05). SEM evaluation at 10,000× magnification (Figure 4a–e) revealed an abundance of bacteria, mainly cocci, adhering onto the surface of all Y‐TZP samples regardless of instrumentation method.

## 4 DISCUSSION

This in‐vitro study was designed to explore the effects of various physical decontamination methods on the surface characteristics of zirconia implant surface and subsequent bacterial adhesion following instrumentation. The results showed that zirconia implant surfaces can be altered based on the type of decontamination method used, although no significant differences in bacterial adhesion was observed.

Four decontamination methods were examined including the use of an US with stainless steel tip, plastic curette, TC and an air abrasive device with glycine powder. The TC, plastic curette and air abrasive device selected for this study were specifically designed and deemed ‘implant safe’ for maintenance procedures. In terms of the US, further investigations were needed to determine their effects on zirconia implants as US with metal tips have been found to damage titanium implants (Harrel et al., 2019; Kawashima et al., 2007). Acid‐etched Y‐TZP discs were used in this study as acid‐etching is a common surface modification technique designed to enhance osseointegration and the effects of instrumentation on acid‐etched zirconia implant surfaces had yet to be explored (Flamant et al., 2016; Hafezeqoran & Koodaryan, 2017). While the exact protocol used by manufacturers to fabricate acid‐etched commercial dental implants is undisclosed, Y‐TZP discs were etched with 40% hydrofluoric acid in accordance with recommendations provided by Flamant et al. (2016). The baseline surface roughness (Sa) value of control acid‐etched Y‐TZP discs in this study was found to be 1.6 μm, which is slightly higher than some commercially available acid‐etched zirconia implants with Sa values ranging from 0.73 to 1.27 μm (Beger et al., 2018). However, according to Albrektsson and Wennerberg (2004), implant surfaces classified as “moderately rough” with a Sa value between 1.0 μm and 2.0 μm may have some clinical advantage over smoother and rougher surfaces due to a stronger bone response.

Previous studies (Checketts et al., 2014; Huang et al., 2019; Lang et al., 2016; Vigolo et al., 2017; Vigolo & Motterle, 2010) have analyzed the topography of zirconia surfaces following decontamination procedures using a variety of surface characterization techniques including profilometry, atomic force microscopy, and SEM. Two‐dimensional surface parameters such as Ra and Rz which measures the surface profile of a single line were also examined in these studies (Checketts et al., 2014; Huang et al., 2019; Lang et al., 2016; Vigolo et al., 2017; Vigolo & Motterle, 2010). In our study, surface characterization was performed using laser scanning microscopy as it analyzes the surface profile over a given area and allows for an accurate assessment of the corresponding 3D surface area parameters Sa, Sz, Ssk, and Sku.

The results of the present study found that plastic curettes and air abrasive devices with glycine powder caused no visible surface alterations (Figure 1c, d). No significant difference in Sa, Sz, Sku, and Ssk was observed in comparison with the control, indicating a preservation of the zirconia surface following instrumentation (Figure 3a–d). These findings are in agreement with previous studies (Huang et al., 2019; Lang et al., 2016; Vigolo & Motterle, 2010) which investigated non‐metal hand instruments and air abrasive devices. The use of an air abrasive device was only examined in one other study (Huang et al., 2019) which found that air abrasion with glycine powder caused no changes to the zirconia surface morphology. Although not examined in this study, Huang et al. (2019) also found minimal surface alterations following treatment of zirconia with carbon‐fiber reinforced plastic curettes. Likewise, Lang et al. (2016) who simulated multi‐year implant maintenance reported a negligible difference in surface roughness on zirconia discs instrumented twenty and one hundred times with plastic curettes. In contrast, Vigolo and Motterle (2010) noted that plastic curettes left behind numerous small scratches on the zirconia surface. A tension load cell was utilized by Vigolo and Motterle (2010) to standardize the amount of pressure applied to be 700 g, which could have led to a higher force application and therefore alterations in the form of scratches.

Gray discolouration of zirconia surfaces following instrumentation was evident in this study with TC and US with stainless steel tips (Figure 1b, e). No significant increase in Sa was noted with TC use, however, the US yielded the greatest Sa value with a significant difference observed in comparison with the control (Figure 3a). Interestingly, the US and TC caused a significant increase in Ssk value (Ssk > 0) indicating the predominance of peaks instead of valley‐like structures within the surface profile (Figure 3d). The prevalence of peaks signifies the deposition of remnants rather than deep grooves or scratches created during instrumentation. The deposition of abraded material from instrument tips was suggested by Checketts et al. (2014) to be a possible reason for metallic marks and unsightly staining of zirconia surfaces after using an US with a metal tip. Consequently, EDS was conducted in the present study to confirm the presence of residual trace elements caused by abrasion of the instruments being used. Chromium and iron, elements commonly present in stainless steel were noted on zirconia surfaces treated with the ultrasonic stainless‐steel tip. Likewise, titanium and barium were present on surfaces treated with the TC. The superior wear resistance and hardness of zirconia as suggested by Huang et al. (2019), relative to the instruments being used may have resulted in instrument degradation rather than surface deterioration. Huang et al. (2019) and Lang et al. (2016) found no significant changes to the surface roughness of zirconia following instrumentation with TC. Conversely, two studies (Vigolo et al., 2017; Vigolo & Motterle, 2010) reported noticeable damage to zirconia surfaces treated with TC and US with metal tips on profilometric and SEM analysis. Hence, the results of the present study suggest that TC and US with stainless steel tips should be used cautiously during decontamination procedures as the metallic residue may compromise the aesthetic appearance of zirconia implants. In addition, the effects of metallic particles on surrounding peri‐implant tissues has not been fully established with some studies suggesting that the presence of metallic particles may influence the pathogenesis of peri‐implant disease and interfere with healing events associated with osseointegration (Fretwurst et al., 2018; Noronha Oliveira et al., 2018; Suarez‐Lopez Del Amo et al., 2018).

Following surface analysis, treated samples were incubated with saliva collected from healthy participants to culture bacterial species normally found in the oral microbiome. The SEM findings of the present study found an abundance of cocci bacteria on all treated zirconia surfaces (Figure 4). No statistically significant difference in bacterial adhesion, as determined by estimation of dislodged bacterial numbers, was found between any of the treatment groups despite the ultrasonically scaled surface having a significant increase in surface roughness compared to that of the control. Conversely, Checketts et al found that stainless steel curettes caused a significant increase in the adherence of *Streptococcus mutans*, *Lactobacillus acidophilus*, and *Actinomyces viscosus* even though there was no significant difference in surface roughness compared to the control (Checketts et al., 2014). Huang et al noted a negligible difference in surface roughness treated with TC, carbon‐fiber reinforced plastic curettes, US with carbon‐fiber tip and air polishing device with glycine powder, however, no difference in bacterial adhesion of *Streptococus mitis* was observed among all treatment groups (Huang et al., 2019). Based on the present and past studies, a direct correlation between decontamination‐induced surface roughness and bacterial adhesion on zirconia surfaces could not be established. This may be due to the influence of other surface factors such as wettability, surface‐free energy and surface chemistry which also affect bacterial adhesion (Teughels et al., 2006).

One of the key limitations of the present study was that the effects of instrumentation were assessed on zirconia discs rather than root form implant fixtures consisting of numerous threads and valleys. In addition, it is difficult to directly quantify bacterial numbers using OD measurements, especially in a salivary biofilm containing a diverse range of different bacteria species. To improve on this study, future studies investigating the effects of instrumentation on the surface topography of implant fixtures rather than the flat surface of zirconia discs are required. In addition, the effects of implant surface changes induced by instrumentation on cellular interactions needs to be explored. Saliva samples collected in peri‐implant pockets would also provide a better representation of microbial species residing around dental implants due to variations in the oral microbiome within different areas of the oral cavity (Kilian et al., 2016). Finally, it remains to be determined how well these instruments perform in clinical practice. The cleaning efficacy of these instruments should be assessed as their effectiveness depends upon their ability to access implant threads within the peri‐implant region.

## 5 CONCLUSION

Within the limitations of this study, air abrasive devices and plastic curettes may be a suitable option for zirconia implant decontamination as minimal surface changes were seen following their use. In contrast, US with stainless steel tips and TC should be used cautiously due to the deposition of metallic remnants on the surface that may present a biological and aesthetic concern. However, further studies are required to clarify the effects of these decontamination methods within the clinical setting.

## CONFLICT OF INTEREST

All authors declare that they have no conflict of interest.

## AUTHOR'S CONTRIBUTIONS

All authors were involved in the conception and design of the study as well as the collection and analysis of the data. NT wrote the draft manuscript which was critically revised by DS, CM, and EA. All authors read and approved the final manuscript for publication.

## Data Availability

The data that support the findings of this study are available from the corresponding author upon reasonable request.

## References

[cre2486-bib-0001] Albrektsson, T. , & Wennerberg, A. (2004). Oral implant surfaces: Part 1–review focusing on topographic and chemical properties of different surfaces and in vivo responses to them. The International Journal of Prosthodontics, 17(5), 536–543. 15543910

[cre2486-bib-0002] Apratim, A. , Eachempati, P. , Krishnappa Salian, K. K. , Singh, V. , Chhabra, S. , & Shah, S. (2015). Zirconia in dental implantology: A review. Journal of International Society of Preventive and Community Dentistry, 5(3), 147–156. 26236672 10.4103/2231-0762.158014 PMC4515795

[cre2486-bib-0003] Atieh, M. A. , Alsabeeha, N. H.M. , Faggion, C. M. & Duncan, W. J. (2012). The frequency of peri‐implant diseases: A systematic review and meta‐analysis. Journal of Periodontology, 1–15. 10.1902/jop.2012.120592 23237585

[cre2486-bib-0004] Beger, B. , Goetz, H. , Morlock, M. , Schiegnitz, E. , & Al‐Nawas, B. (2018). In vitro surface characteristics and impurity analysis of five different commercially available dental zirconia implants. International Journal of Implant Dentistry, 4(1), 13. 29696470 10.1186/s40729-018-0124-8 PMC5918143

[cre2486-bib-0005] Caton, J. G. , Armitage, G. , Berglundh, T. , ILC, C. , Jepsen, S. , Kornman, K. S. , Mealey, B. L. , Papapanou, P. N. , Sanz, M. , & Tonetti, M. S. (2018). A new classification scheme for periodontal and peri‐implant diseases and conditions: Introduction and key changes from the 1999 classification. Journal of Clinical Periodontology, 20(S20), S1–S8. 10.1111/jcpe.12935 29926489

[cre2486-bib-0006] Checketts, M. R. , Turkyilmaz, I. , & Asar, N. V. (2014). An investigation of the effect of scaling‐induced surface roughness on bacterial adhesion in common fixed dental restorative materials. The Journal of Prosthetic Dentistry, 112(5), 1265–1270. 24831748 10.1016/j.prosdent.2014.04.005

[cre2486-bib-0007] Dosumu, O. O. , Ogunrinde, J. T. , & Bamigboye, S. A. (2014). Knowledge of consequences of missing teeth in patients attending prosthetic clinic in u.C.h. Ibadan. Annals of Ibadan Postgraduate Medicine, 12(1), 42–48. 25332700 PMC4201933

[cre2486-bib-0008] Flamant, Q. , Marro, F. G. , Rovira, J. J. R. , & Anglada, M. (2016). Hydrofluoric acid etching of dental zirconia. Part 1: Etching mechanism and surface characterization. Journal of the European Ceramic Society, 36(1), 121–134. 10.1016/j.jeurceramso

[cre2486-bib-0009] Fretwurst, T. , Nelson, K. , Tarnow, D. P. , Wang, H. L. , & Giannobile, W. V. (2018). Is metal particle release associated with peri‐implant bone destruction? An emerging concept. Journal of Dental Research, 97(3), 259–265. 29130804 10.1177/0022034517740560

[cre2486-bib-0010] Grech, J. , & Antunes, E. (2019). Zirconia in dental prosthetics: A literature review. Journal of Materials Research and Technology, 8(5), 4956–4964.

[cre2486-bib-0011] Grech, J. , & Antunes, E. (2020). Optimization of two‐step sintering conditions of zirconia blanks for dental restorations. Ceramics International, 46(16), 24792–24798. 10.1016/j.ceramint.2020.07.068

[cre2486-bib-0012] Guglielmotti, M. B. , Olmedo, D. G. , & Cabrini, R. L. (2019). Research on implants and osseointegration. Periodontology 2000, 79(1), 178–189. 30892769 10.1111/prd.12254

[cre2486-bib-0013] Gulati, M. , Govila, V. , Anand, V. , & Anand, B. (2014). Implant maintenance: A clinical update. International Scholarly Research Notices, 2014, 1–8. 10.1155/2014/908534 PMC4897104 27437506

[cre2486-bib-0014] Hafezeqoran, A. , & Koodaryan, R. (2017). Effect of zirconia dental implant surfaces on bone integration: A systematic review and meta‐analysis. BioMed Research International, 2017, 9246721. 28299337 10.1155/2017/9246721 PMC5337335

[cre2486-bib-0015] Harrel, S. K. , Wilson, T. G., Jr. , Pandya, M. , & Diekwisch, T. G. H. (2019). Titanium particles generated during ultrasonic scaling of implants. Journal of Periodontology, 90(3), 241–246. 30312471 10.1002/JPER.18-0230

[cre2486-bib-0016] Huang, Y. S. , Hung, C. Y. , & Huang, H. H. (2019). Surface changes and bacterial adhesion on implant abutment materials after various clinical cleaning procedures. Journal of the Chinese Medical Association, 82(8), 643–650. 31305347 10.1097/JCMA.0000000000000139 PMC13048108

[cre2486-bib-0017] Kawashima, H. , Sato, S. , Kishida, M. , Yagi, H. , Matsumoto, K. , & Ito, K. (2007). Treatment of titanium dental implants with three piezoelectric ultrasonic scalers: An in vivo study. Journal of Periodontology, 78(9), 1689–1694. 17760537 10.1902/jop.2007.060496

[cre2486-bib-0018] Khan, A. , & Sharma, D. (2020). Management of peri‐implant diseases: A Survey of Australian Periodontists. Dentistry Journal, 8(3), 100. 10.3390/dj8030100 PMC7558189 32882900

[cre2486-bib-0019] Khan, A. , Goyal, A. , Currell, S. D. , & Sharma, D. (2020). Management of peri‐implantitis lesions without the use of systemic antibiotics: A systematic review. Dentistry Journal, 8(3), 106. 10.3390/dj8030106 PMC7576475 32937892

[cre2486-bib-0020] Kilian, M. , Chapple, I. L. , Hannig, M. , Marsh, P. D. , Meuric, V. , Pedersen, A. M. L. , Tonetti, M. S. , Wade, W. G. , & Zaura, E. (2016). The oral microbiome ‐ an update for oral healthcare professionals. British Dental Journal, 221(10), 657–666. 27857087 10.1038/sj.bdj.2016.865

[cre2486-bib-0021] Kim, K. T. , Eo, M. Y. , Nguyen, T. T. H. , & Kim, S. M. (2019). General review of titanium toxicity. International Journal of Implant Dentistry, 5(1). 10.1186/s40729-019-0162-x PMC6409289 30854575

[cre2486-bib-0022] Klinge, B. , Klinge, A. , Bertl, K. , & Stavropoulos, A. (2018). Peri‐implant diseases. European Journal of Oral Sciences, 126(S1), 88–94. 10.1111/eos.12529 30178555

[cre2486-bib-0023] Lang, M. S. , Cerutis, D. R. , Miyamoto, T. , & Nunn, M. E. (2016). Cell attachment following instrumentation with titanium and plastic instruments, diode laser, and titanium brush on titanium, titanium‐zirconium, and zirconia surfaces. The International Journal of Oral & Maxillofacial Implants, 31(4), 799–806. 27447145 10.11607/jomi.4440

[cre2486-bib-0024] Louropoulou, A. , Slot, D. E. , & Van der Weijden, F. (2015). Influence of mechanical instruments on the biocompatibility of titanium dental implants surfaces: A systematic review. Clinical Oral Implants Research, 26(7), 841–850. 24641774 10.1111/clr.12365

[cre2486-bib-0025] Louropoulou, A. , Slot, D. E. , & Van der Weijden, F. A. (2012). Titanium surface alterations following the use of different mechanical instruments: A systematic review. Clinical Oral Implants Research, 23(6), 643–658. 21564303 10.1111/j.1600-0501.2011.02208.x

[cre2486-bib-0026] Munro, T. , Miller, C. M. , Antunes, E. , & Sharma, D. (2020). Interactions of osteoprogenitor cells with a novel zirconia implant surface. Journal of Functional Biomaterials, 11(3), 50. 10.3390/jfb11030050 PMC7565437 32708701

[cre2486-bib-0027] Noronha Oliveira, M. , Schunemann, W. V. H. , Mathew, M. T. , Henriques, B. , Magini, R. S. , Teughels, W. , & Souza, J. C. M. (2018). Can degradation products released from dental implants affect peri‐implant tissues? Journal of Periodontal Research, 53(1), 1–11. 28766712 10.1111/jre.12479

[cre2486-bib-0028] Özkurt, Z. , Kazazoğlu, E. (2011). Zirconia dental implants: A literature review. Journal of Oral Implantology, 37(3), 367–376. 10.1563/aaid-joi-d-09-00079 20545529

[cre2486-bib-0029] Park, J. B. , Lee, S. H. , Kim, N. , Park, S. , Jin, S. H. , Choi, B. K. , Kim, K. K. , & Ko, Y. (2015). Instrumentation with ultrasonic scalers facilitates cleaning of the sandblasted and acid‐etched titanium implants. The Journal of Oral Implantology, 41(4), 419–428. 24552131 10.1563/AAID-JOI-D-13-00078

[cre2486-bib-0030] Renvert, S. , Hirooka, H. , Polyzois, I. , Kelekis‐Cholakis, A. , Wang, H. L. , & Working, G. (2019). Diagnosis and non‐surgical treatment of peri‐implant diseases and maintenance care of patients with dental implants – Consensus report of working group 3. International Dental Journal, 69(Suppl 2), 12–17. 31478575 10.1111/idj.12490 PMC9379037

[cre2486-bib-0031] Sargozaie, N. , Moeintaghavi, A. , & Shojaie, H. (2017). Comparing the quality of life of patients requesting dental implants before and after implant. The Open Dentistry Journal, 11, 485–491. 29114333 10.2174/1874210601711010485 PMC5646019

[cre2486-bib-0032] Sivaraman, K. , Chopra, A. , Narayan, A. I. , & Balakrishnan, D. (2018). Is zirconia a viable alternative to titanium for oral implant? A critical review. Journal of Prosthodontic Research, 62(2), 121–133. 28827030 10.1016/j.jpor.2017.07.003

[cre2486-bib-0033] Stanford CM . Dental implants. A role in geriatric dentistry for the general practice? The Journal of the American Dental Association. 2007;138, 34S‐40S. 17761844 10.14219/jada.archive.2007.0361

[cre2486-bib-0034] Suarez‐Lopez Del Amo, F. , Garaicoa‐Pazmino, C. , Fretwurst, T. , Castilho, R. M. , & Squarize, C. H. (2018). Dental implants‐associated release of titanium particles: A systematic review. Clinical Oral Implants Research, 29(11), 1085–1100. 30280418 10.1111/clr.13372

[cre2486-bib-0035] Teughels, W. , Van Assche, N. , Sliepen, I. , & Quirynen, M. (2006). Effect of material characteristics and/or surface topography on biofilm development. Clinical Oral Implants Research, 17(Suppl 2), 68–81. 16968383 10.1111/j.1600-0501.2006.01353.x

[cre2486-bib-0036] Vigolo, P. , Buzzo, O. , Buzzo, M. , & Mutinelli, S. (2017). An in vitro evaluation of alumina, zirconia, and lithium disilicate surface roughness caused by two scaling instruments. Journal of Prosthodontics, 26(2), 129–135. 26683122 10.1111/jopr.12424

[cre2486-bib-0037] Vigolo, P. , & Motterle, M. (2010). An in vitro evaluation of zirconia surface roughness caused by different scaling methods. The Journal of Prosthetic Dentistry, 103(5), 283–287. 20416411 10.1016/S0022-3913(10)60059-5

[cre2486-bib-0038] Yeo, I. S. , Kim, H. Y. , Lim, K. S. , & Han, J. S. (2012). Implant surface factors and bacterial adhesion: A review of the literature. The International Journal of Artificial Organs, 35(10), 762–772. 23138699 10.5301/ijao.5000154

